# Diabetic nephropathy: What does the future hold?

**DOI:** 10.1007/s11255-015-1121-y

**Published:** 2015-10-05

**Authors:** R. M. Montero, A. Covic, L. Gnudi, D. Goldsmith

**Affiliations:** Renal, Dialysis and Transplantation Unit, Guy’s and St Thomas’ Hospital, London, UK; Hospital “C.I.Parhon” and University of Medicine “Grigore T Popa”, Iasi, Romania; Cardiovascular Division, Department of Diabetes and Endocrinology, Guy’s and St Thomas’ Hospital, School of Medicine and Life Science, King’s College London, London, UK

**Keywords:** Diabetic nephropathy, RAAS blockade, Albuminuria, Inflammation, Anti-fibrotics, New therapies

## Abstract

The consensus management of diabetic nephropathy (DN) in 2015 involves good control of glycaemia, dyslipidaemia and blood pressure (BP). Blockade of the renin–angiotensin–aldosterone system using angiotensin-converting enzyme inhibitors, angiotensin-2 receptor blockers or mineralocorticoid inhibitors are key therapeutic approaches, shown to be beneficial once overt nephropathy is manifest, as either, or both, of albuminuria and loss of glomerular filtration rate. Some significant additional clinical benefits in slowing the progression of DN was reported from the Remission clinic experience, where simultaneous intensive control of BP, tight glycaemic control, weight loss, exercise and smoking cessation were prioritised in the management of DN. This has not proved possible to translate to more conventional clinical settings. This review briefly looks over the history and limitations of current therapy from landmark papers and expert reviews, and following an extensive PubMed search identifies the most promising clinical biomarkers (both established and proposed). Many challenges need to be addressed urgently as in order to obtain novel therapies in the clinic; we also need to examine what we mean by remission, stability and progression of DN in the modern era.

## Introduction: obesity, diabetes and CKD—a cardio-metabolic tsunami

The rapidly rising incidence of diabetes mellitus (DM) worldwide is reputed to impact over 380 million people [1]. Table 1 shows the current and predicted incidences of chronic kidney disease (CKD), DM, and patients with DM that have reached end-stage kidney disease (ESKD). China now has the highest rates of number of people with DM, around 98.4 million, followed by India with 65.1 million and then the USA [2].

**Table 1:** Predictions of population incidences of USA and UK [6–13]

Worldwide DM	1980–1990	2010–2020
	153 million	472 million
*USA*		
CKD	19 million	>26 million
DM	5.8 million	24 million
ESKD with DM	17,727	48,215
*UK*		
CKD	1.7 million	3.5 million
DM	2.9 million	5 million
ESKD with DM	870,000	1.7 million
*Europe*		
CKD	59.3 million	65.9 million
DM	66.8 million	68.9 million
ESKD with DM	6.6 million	6.8 million

DM is the fifth cause of morbidity and mortality worldwide [3], and the most common cause of ESKD in the Western world [4]. The European Renal Association (ERA-EDTA) registry data showed that ten countries across Europe had an increase of 11.9 % per year of type 2 DM (T2DM) patients starting renal replacement therapy (RRT) [5].

T2DM increasingly arises in a younger and more obese population with metabolic syndrome [14] whose natural history is currently unknown but predicted to lead to full insulin resistance with a decline in renal function leading to CKD with proteinuria. The increasing rates of global obesity are a major drive in the development of diabetes, CKD and cardiovascular disease (CVD), representing a major health and health-economic burden to the developed and developing worlds. However, a significant proportion of patients with DM and CKD stage 3a/3b do not progress to ESKD and either stabilise with their current treatment therapies or die from their significantly higher cardiovascular mortality risks before RRT is needed [4, 15]. Analysis of the baseline characteristics of the Study of Heart and Renal Protection (SHARP) study showed the importance of primary renal disease leading to CKD [16]. The highest mortality overall is seen in patients with DM with a peak in mortality occurring prior to reaching ESKD.

The UK renal registry reported a mortality of 30 % in DM on RRT aged 18–44 years at 5 years: much higher than the 11 % observed in non-diabetic patients [17]. Following 5 years of RRT, 34 % of non-diabetic patients aged 45–64 died compared with 51 % in the DM group. The median life expectancy in non-diabetic patients requiring RRT aged <45 years was reported by the UK renal registry as 9 years more than in patients with DM of the same age. Thus, a further decade of life lost in this group of patients, many of whom, because of associated co-morbidities, are not promising candidates for pancreas and/or renal transplants.

Mahmoodi’s meta-analysis of >1 million people showed an association between all-cause and cardiovascular mortality and CKD in non-hypertensive and hypertensive people with low GFRs and raised albumin–creatinine ratio (ACR). Patients with CKD alone had an all-cause and cardiovascular mortality, respectively, of 4.1 and 0.9 %, while those with CKD and hypertension had an all-cause and cardiovascular mortality, respectively, of 15 and 6.8 %. A subsequent meta-analysis looking at CKD with or without diabetes found similar relative risks of mortality between these two groups, thereby emphasising the importance of CKD as a major driver for mortality in these populations [18].

The degree of albuminuria has long been used to determine progression of DN with Adler reporting an annual incidence of people with diabetes progressing from normoalbuminuria to microalbuminuria (2.0 %/year) to macroalbuminuria (2.8 %/year) and to ESKD (2.3 %/year) [19]. This is otherwise known as the “classical progression paradigm”, with mortality increasing at each stage (3.0, 4.6, 19.2 %/year, respectively). The decline in GFR has previously been thought to mirror changes in albuminuria in DN; however, there are increasing reports that the decline in GFR may occur irrespective of the degree of albuminuria in a non-proteinuric DN phenotype [20]. It is, however, well established that the presence of albuminuria and GFR independently and additively contribute to the cardiovascular and renal risk [21]. The alarming rates of disease incidence and progression continue despite the deployment of all current treatments and while achieving, in many cases, significant reductions in proteinuria require us to re-look at this well-known disease.

## The basic challenge: treating the whole patient by looking beyond renal endpoints

### The pathophysiology, history, effects and limitations of RAAS blockade

**Fig. 1:**
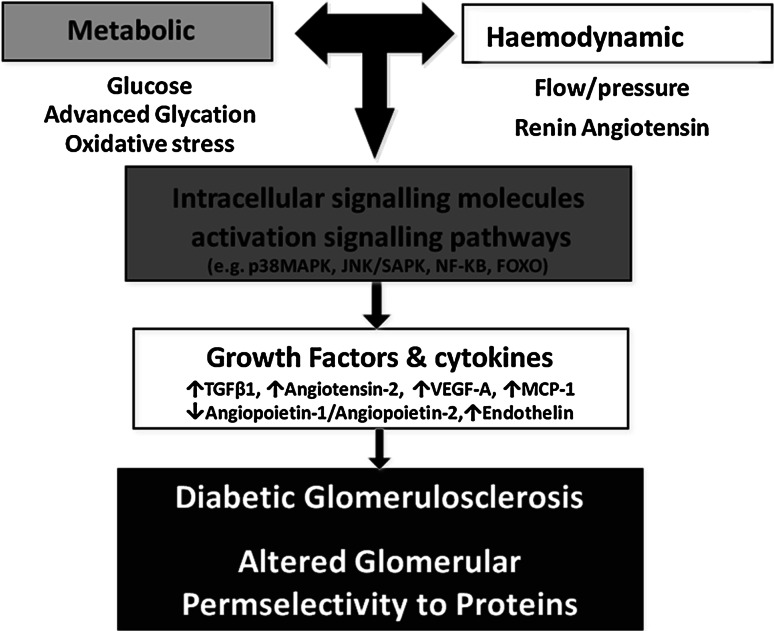
Pathophysiology of DN

The mechanisms behind the pathophysiology of DN are complex and continue to be incompletely understood. Both metabolic (hyperglycaemia) and haemodynamic perturbations interact synergistically [22, 23] and have been reported to activate local RAAS resulting in increased angiotensin-2, reactive oxygen species (ROS), inflammation, expression of transforming growth factor-β (TGF-β) and dysregulation of different vascular growth factors such as the VEGF-A (upregulated in the initial phase of the disease) and the angiopoietin/Tie-2 system (with excess of angiopoietin-2 over angiopoietin-1) [24] (Fig. 1). This would otherwise be known as the classical pathway of DN where the hallmark features of thickening of the glomerular basement membrane, mesangial expansion and glomerulosclerosis are classically seen on renal biopsy [25] in combination with albuminuria and GFR decline. Mauer had described these renal biopsy changes in 285 people with T1DM with normoalbuminuria who had been given enalapril, losartan or placebo. In this study, formal GFRs and a repeat renal biopsy were undertaken to determine any histological changes between those on different ACEi/ARB treatments. This small study showed the decline in GFR and the change in mesangial fractional volume of each glomerulus determining the degree of mesangial expansion. They found the changes were comparable in all groups [26], and thus, albuminuria was insensitive to these changes.

The risk of renal biopsy has prevented further larger studies performing this procedure on those without proteinuria, and hence, it is unclear when the classical underlying histological changes occur or alter with renal function decline or proteinuria. The success of a DN animal model is that which has developed these histological features and whereby treatments act to reverse them; however, the problem therein lies that the existing models of DN do not develop albuminuria or have this without the histological features seen in man. Interestingly, advanced renal damage has been reported in a study looking at nephrectomised cancer renal biopsies in those with T2DM without overt proteinuria suggesting that these changes may occur with GFR decline and worsen with the presence of proteinuria [20]. Other studies have attempted to reassess the features seen on the DN renal biopsy, and an increased heterogeneity within the histology of those with DN has been reported with the severity of glomerulopathy, tubulointerstitial fibrosis and arteriolar hyalinosis in those with microalbuminuria quantified in the Tervaert’s histopathological classification [25, 27, 28]. This clearly illustrates that the simple paradigm of progression once depicted is far more complex than initially thought.

More recently, there has been appreciation for the role of inflammation in the progression of DN. The mechanisms employed are poorly understood with reports of an increase in inflammatory cytokines (MCP-1, TNF-α) and mononuclear infiltrates in the glomeruli and tubulointerstitium that contribute to the progression of DN [29, 30]. Peroxisome proliferator-activated receptors-γ (PPAR-γ) (glitazones) agonists have been reported to be reno-protective in experimental model of diabetes and in people with diabetes via their anti-inflammatory actions, in a metabolic independent manner [31, 32].

The RAAS has multiple roles in promoting progression of all renal diseases, particularly in DN [33]. The blockade of RAAS in animal and in vitro studies have shown a down-regulation of TGF-β, AGE, ROS, NADPH oxidase, RAGE expression and collagen IV (Col4) production—all leading to a reduction in mesangial expansion, glomerulosclerosis, inflammation and tubulointerstitial fibrosis [34–39]. These findings have further fuelled the desire to block RAAS as the sole treatment for DN.

Numerous studies have demonstrated since the 1980s how good blood pressure (BP) and strict glycaemic control decrease cardiovascular mortality in patients with DM and renal impairment [4, 19, 40]. The treatment of patients with DM with CKD was revolutionised in the 1990s, with most cited landmark papers such as the Captopril study in type 1 DM (T1DM) reporting the loss of creatinine of 11 % from 84 ml/min (~9 ml/min/year) versus 17 % from 79 ml/min (~13 ml/min/year) without captopril [41]. The Reduction of Endpoints in Non-insulin-dependent DM in the Angiotensin-2 Antagonist Losartan study (RENAAL) [42] simultaneously published with the Irbesartan Diabetic Nephropathy Trial (IDNT) both showed a decrease in GFR decline or a delay in the doubling of creatinine and a reduction in proteinuria in the ARB arm [43]. The possibility of preventing renal progression with eliminating this residual proteinuria with further agents suggested a further benefit by achieving complete RAAS blockade.

#### Earlier stage DN: normoalbuminuria to proteinuria, microalbuminuria to proteinuria

A delay in progression from microalbuminuria to overt proteinuria was described since 1995 with earlier studies focused on determining the significance of increasing proteinuria [44, 45]. Subsequently, Perkins showed factors associated with regression rather than progression of proteinuria. The DCCT/EDIC study showed 28 % of microalbuminurics became macroalbuminuric with 15 % sustaining a loss of GFR and 4 % leading onto ESKD [46]. The introduction of RAAS blockade reduced proteinuria; regression rates to normoalbuminuria were 40 %, thus emphasising the relative insensitivity of albuminuria as a marker of progression in DN [47].

Undeniably, GFR decline occurs more rapidly in the presence of overt or nephrotic range proteinuria [48]; however, there has been a lack of appreciation of the degree of GFR decline occurring with minimal albuminuria. The loss of GFR is increasingly being reported in diabetic patients with normoalbuminuria, again illustrating how treatments targeting the resolution of albuminuria may already be too late in the disease or not work at all [49, 50]. In addition, formulae used to determine GFR are not sensitive at levels of GFR >90 ml/min. The limitations of this formula are also apparent within the acute kidney injury arena where people with normal renal function without proteinuria undergo a substantial rapid decline in renal function secondary to an insult that may range from sepsis to drug induced that is not identified until the decline reaches levels the formula identifies as low [51]. The modification of diet in renal disease (MDRD) formula has been established as having a higher sensitivity in GFR <60 ml/min with the chronic kidney disease epidemiology (CKD-EPI) formula reported to be more sensitive at higher GFRs; however, fundamentally the formulae are dependent on creatinine as the gold standard sensitive marker of renal injury. This marker, however, is non-specific to the type of histological injury. The laborious iohexol/inulin methods are employed in cases where true GFR is fundamental in providing drug treatments such as chemotherapy or for donors prior to living kidney donation [52] and may detect decline in renal function; however, these are seldom used to assess the renal function in those with DN.

The introduction of RAAS blockade at this early stage of DN may not provide any benefit and emphasises the urgent need for more sensitive markers to detect early stage DN. This also challenges the simple paradigm of albuminuria leading to GFR loss and steady progression that may be conceptually too simplistic for such a complex disease.

### Combining RAAS blockade with other, mainly cardioprotective, interventions

Dual blockade of RAAS was done using different agents in a number of studies with differing effects on the surrogate endpoint trials have adopted (Table 2). The safety of dual blockade was brought into question in these studies where new therapies are additive to existing ACEi/ARB therapy. The use of this combination in T2DM patients continues to be controversial and currently is not recommended. The effects of aliskiren alone have not been assessed as trials conducted with new agents currently act as adjuncts to RAAS blockade rather than alternatives to ACEi/ARB.

**Table 2:** Outcome of landmark studies with RAAS blockade

Trial	AVOID [53]	ALTITUDE [50]	VA NEPHRON D [54]	Aldosterone antagonists [55]
Agent	Aliskiren	Aliskiren	ACEi (Lisinopril)	Aldosterone antagonist
Combined ACEi/ARB	ARB (Losartan)	ARB (Irbesartan)	ARB (Losartan)	ACEi/ARB (Lisinopril/Losartan)
Reduction of blood pressure	Yes	Yes	Yes	Yes
Decrease in the reduction in GFR	Yes (−2.4 mls/min)	Trial terminated	No effect	Yes (13 % less than placebo)
Anti-proteinuric effect	Yes	Yes	Yes	Yes
Effect on CV mortality	No effect	Increased events	No effect	Not powered
Hyperkalaemic events	Same as placebo	Increased	Increased	Increased
Acute kidney injury	Same as placebo	Increased	Increased	Same as placebo
Progression to ESKD	No effect	Trial terminated	No effect	Not powered
Number of patients	599	8561	1448	81

### Intensification of therapies with RAAS blockade

The importance of a multistrategy approach with RAAS blockade led to the Steno-2 study [56], whereby intensive glycaemic and BP control was achieved. Smoking cessation, weight loss and a reduction in dietary sodium intake were additionally maintained. The results supported a multifactorial approach [56, 57] to achieve lower cardiovascular (CV) events and mortality in the intensely monitored group after 13.3 years [58]. A multimodal strategy approach to the management of DN is supported by the Remission clinic [59]. Dual RAAS inhibition was employed with intensified BP control, statin use, smoking cessation and implementation of a healthy lifestyle. The median follow-up was 4 years and suggested that a multidrug approach in the treatment of CKD could slow the progression of CKD in T2DM and non-diabetics, similar to the Steno-2 study [56]. The Remission clinics had a median decline of −0.17 versus −0.56 ml/min/1.73 m^2^/year of GFR in the intensive versus conventional treatment groups. BP decreased and a 50 % decline in urinary proteinuria from baseline levels was seen. Intensive monitoring avoided a rise in hyperkalaemic events supporting the use of this treatment strategy in T2DM with hypertension and normoalbuminuria, similar to the Bergamo Nephrologic Diabetes Complications Trial (BENEDICT); however, all the patients in the BENEDICT trial were hypertensive to begin with [60]. The BENEDICT trial initially showed that trandolapril delayed the onset of microalbuminuria in T2DM with normoalbuminuria and hypertension.

Previous studies suggest a reduction in CV endpoints and mortality in all patients with diabetes adhering to a diastolic BP of <80 mmHg [61–63]. Whether a systolic BP of <130 or <140 mmHg has additional benefits on CVD and mortality remains unknown [61]. Systolic BP <125 mmHg was reported to be deleterious in the MDRD study and in diabetic patients >70 years of age [64–66]. To achieve systolic BP <130 mmHg requires an intense approach seen in both the Remission and Steno-2 single-centre approaches. Despite considerable efforts, few patients managed smoking cessation or regular exercise in the Steno-2 study. The Steno-2 study also supplemented glycaemic and BP control with dietary supplements of vitamins E and C together with folic acid, which is not standard clinical practice. These approaches, however, (Steno, Bergamo) are completely impractical for normal clinical practice due to the intensity required to manage the needs of an ever-growing population.

## The next challenge: defining renal endpoints and how to measure them

Traditionally, a change in proteinuria, a doubling of serum creatinine or reaching ESKD are the renal endpoints used to determine outcome and efficacy of treatment in renal trials (Table 3).

**Table 3:** Current renal endpoints used in trials

Trial	Primary renal endpoints	Effects on ACR	Effects on GFR
PREVEND (Prevention of Renal and Vascular End-stage Disease Intervention trial)	CV mortality, hospitalisation for CV morbidity and reaching ESKD	Decrease	No change
RENAAL (see above)	CV mortality, time to ESKD	Decrease	Decrease GFR slowed with ARB
IDNT (see above)	Double serum creatinine	Decrease	Decrease GFR slowed with ARB
BEACON (see below)	Decrease risk of ESKD or CV mortality in T2DM with CKD IV	Increase	Increase in GFR

These traditional “biomarkers” have several limitations (see above) and require large and costly interventional trials. The trial endpoints currently used require long trials to assess the CV mortality and whether a progression of ESKD or a double of serum creatinine is reached in this variably progressive disease. Using these trial endpoints limits, the ability to determine treatment effect with no insight into how the natural history of the disease and its complications are affected, due to the longer time point surrogate markers employed. This has led to suggestions of measuring other proteins in new clinical trials to compare this with albuminuria [50]. To assess whether the change or persistence in albuminuria continues despite the new intervention and compares these effects with the different severity of disease within the chosen population. It has also been suggested that an alternative surrogate endpoint could be a 30–40 % decrease in GFR with albuminuria defined as new onset, progression or regression with micro-/macroalbuminuria, however, discussion in this area continue [67]. Development of new biomarkers looking at different underlying structural damage or pathophysiological pathways opens new avenues for assessing progressive DN (Table 4); however, how these biomarkers correlate with human histopathological changes is currently unknown and not routinely practiced.

**Table 4:** Biomarkers for DN

Mechanism	Potential biomarkers
Glomerular	Cystatin C Urinary Col4, transferring, caeruloplasmin, MCP-1
Tubuloepithelial	NAG, NGAL, KIM-1 Urinary α1-microglobulin, L-FABP, CTGF, RBP
Oxidative stress and inflammation	Urinary 8-OHdG Serum TNFR, IL-6, TNFα Urinary pentosidine, TNFα, α1 acid glycoprotein, VEGF, YKL-40
Endothelial dysfunction	Tyrosine kinase C-type natriuretic peptide
Genomics, proteomics and metabolomics	AFF3 gene CKD273
microRNAs	Urinary or plasma miR192, miR29

### Biomarkers in DN: Better than albuminuria or GFR?

The limitations of using albuminuria or GFR alone are apparent with their use as trial endpoints requiring further thought. New biomarkers also have their limitations and may be perceived as no better than those established, however, using more specific markers that are specific to underlying glomerular or tubular dysfunction, the different stages of disease progression may be identified and used to eloquently individualise therapies.

#### Glomerular damage

Cystatin C is a 13.3-kDa freely filtered plasma protein that is catabolised by tubular cells, thereby not returning to the circulation and has been described as a more sensitive marker of renal function than serum creatinine in early DN [68, 69]. This marker could be adopted for trials looking at early DN where GFR is less sensitive.

Urinary and serum Col4 with a low molecular weight (LMW) of 540 kDa are elevated in DN [70]. Raised urinary Col4 levels occur in normoalbuminuric DN and could be an earlier marker than microalbuminuria of underlying glomerular damage [71]. However, Col4 levels decrease with improved glycaemic control, limiting its use [72]. In contrast, urinary transferrin (76.5 kDa) is a plasma protein that increases independently of albuminuria [73] and a predictor of development of microalbuminuria in normoalbuminuric T2DM irrespective of BP or HbA1c [74]. This may be a useful marker in CKD 3a disease or to determine underlying hyperfiltration.

Urinary monocyte chemoattractant protein-1 (MCP-1/CCL2) increases in macroalbuminuric but not in microalbuminuria T2DM patients [75]. Urinary MCP-1 levels have been shown to correlate with DN tubulointerstitial lesions, fibrosis and glomerular injury [76, 77]. This biomarker has the potential to determine progressors, and in trials looking at microalbuminuria, detection of this could be used as a new endpoint of predicting ongoing disease. Raised urinary caeruloplasmin (132 kDa) is an acute-phase protein whose excretion increases in normoalbuminuric DN, but its usefulness is limited by its reversibility with improved glycaemic control [78]. Matix metalloproteinases (MMP9) are reported to be increased in DN where the ECM has broken down and there is increased cell turnover. MMP9 is also raised during podocyte dedifferentiation and may be used as an early marker of DN [79].

#### Tubuloepithelial makers

Trials measuring biomarkers with different underlying histopathologies will help develop a greater understanding of the occurrence of these processes and landmark disease processes. This will allow treatments to be specifically targeted to the underlying pathways leading to these changes and allow them to be adapted and personalise the individuals, therapy to prevent progression. Tubuloepithelial markers in combination with glomerular markers may result in a reasonable predictive trial surrogate endpoint.

An increase in secretion or decrease in reabsorption of LMW plasma proteins may be detected in the urine reflecting tubuloepithelial cell damage. *N*-acetyl-β-d-glucosaminidase (NAG) is a 140-kDa lysosomal enzyme in renal tubules that increases in normoalbuminuric DN with significantly raised levels occurring with transition from micro- to macroalbuminuria in T2DM [80]. Neutrophil gelatinase-associated lipocalin (NGAL) (25-kDa) protein stored in neutrophilic granules bind and transport small hydrophobic molecules to regulate the immune system and apoptosis [81]. Raised levels of NGAL are seen in the hyperfiltration stage of DN with a lowering of GFR and a raised cystatin C when compared with healthy controls [82]. When adjusted for BP, HbA1c and duration of diabetes, the difference is no longer significant. Kidney injury molecule-1 (KIM-1) is a type-1 cell membrane glycoprotein on the apical membrane of the proximal tubule that increases with tubular damage in DN [83]. Hyper-filtration in T2DM results in raised urinary levels of KIM-1; however, these are decreased with RAAS blockade limiting its use to a marker of active tubular injury [84].

Urinary connective tissue growth factor (CTGF) and TGF-β are increased in micro- and macroalbuminuric DN [85, 86] and may reflect the occurrence of underlying fibrosis that may be used as a marker for disease progression; 15-kDa intracellular carrier protein liver-type fatty acid binding protein (L-FABP) found in the liver and proximal tubular cells is a marker of structural and functional renal tubular damage. This increases with diabetes and albuminuria [87]. Again, its use is limited with the use of RAAS blockade. In contrast, urinary retinal binding protein (RBP), a 21-kDa freely filtered microprotein that is almost completely reabsorbed in the proximal tubules, is a highly sensitive marker of early DN with its excretion reflecting damage in normoalbuminuria [88]. RBP has also been correlated with micro- and macrovascular complications of T2DM. Studies suggest urinary α1-microglobulin (A1M), a 26-kDa microprotein similar to RBP, may be used to determine tubular dysfunction in early DN [89].

#### Markers of oxidative stress and inflammation

ROS are produced with hyperglycaemia and contribute to diabetic complications via the accumulation of sorbitol and the formation of advanced glycation end products (AGE) and activation of protein kinase C pathways. Enzyme cleavage of ROS-induced 8-hydroxylation of guanine base from nuclear and mitochondrial DNA results in 8-oxo-7, 8-dihydro-2-deoxyguanosine (8-OHdG) as a product of oxidative DNA damage in the urine [90]. These urinary levels may be used to determine early underlying oxidative stress damage.

Serum and urinary TNFα levels increase with micro- and microalbuminuria in T2DM [91]. TNF receptors (TNFRs) 1 and 2 mediate the effects of TNFα. Serum levels of these membrane bound receptors correlate with GFR in DN independent of albuminuria [92]. Raised baseline serum TNFR in patients with DM has predicted progression of CKD 3–5 independently of albuminuria after 12 years [93, 94] and is a promising biomarker of early DN.

Serum interleukin-6 (IL-6) increases with albuminuria and in those with underlying glomerular basement membrane thickening [95, 96]. Urinary orosomucoid or α1 acid glycoprotein (AGA) is a polypeptide released in response to inflammatory cytokines IL-6 and TNFα and reported to be raised in CVD, lung cancer and DM [97]. Urinary levels of vascular endothelial growth factor (VEGF) (an angiogenic inducing cytokine related to endothelial permeability) are increased in normoalbuminuric DN [98].

#### Endothelial dysfunction

YKL-40 is a pro-inflammatory marker independently associated with albuminuria in early T2DM [99, 100]. High levels have been reported to predict CV mortality [101]. Tyrosine kinase is predominantly found in endothelial cells and has been described as a biomarker for underlying angiogenesis pathways [102]. It binds to angiopoietin of endothelial cells to induce angiogenesis. C-type natriuretic peptide (CNP) arises from damage to the vascular endothelium and is a predictor of GFR decline [103].

#### Genomics, proteomics and metabolomics

The development of DN arises from a combination of genetic and environmental factors. Genome-wide association scans have been widely used to determine gene variants predisposing to DN [104]. AFF3 gene on chromosome 15 has been described in T1DM with DN and also seen as an inducer of TGF-β fibrosis in cultured epithelial cells [105]. By contrast, proteomics will provide multiple markers of DN combining cytokines, collagen fragments and RBP [106–108]. A panel of 273 urinary peptides (CKD273) was reported to be specific and sensitive for the early detection of DN [109]. The risk of progression was identified with this panel in a cohort of 35 patients with DM and normoalbuminuria followed up for 5 years [110]. CKD273 has subsequently been validated in a multicentre study that distinguished DN from controls with a factor cut-off, thereby determining individuals at a high risk of progressing [110, 111].

There is a paucity of studies in metabolomics whereby the measure of cellular function using LMW intermediate and end products of the cell reflect the genomic, transcription and proteomic function of the cell affected by the underlying disease pathology [112]. Changes have been detected in the phospholipid and amino acid metabolism of normoalbuminuric diabetic patients and healthy controls [113]. Consideration of the benefit of renal biopsy in trial patients to assess the effect of treatment and that of disease on the biomarkers used may need to be taken prior to determining the accuracy of these markers to reflect underlying disease processes in man.

#### MicroRNAs

MicroRNAs (miR) are short non-coding RNAs of 20–22 nucleotide length that may be measured in plasma and urine and use posttranscriptional mechanisms to result in gene silencing via the reduction of translation or induction of target degradation [114, 115]; 60 % of the human protein coding genes are thought to be regulated by miR [116]. miR192 upregulates miR216a/217 and miR200b/c that increases collagen expression stimulated by TGF-β miR-mediated mechanism. This may promote autoregulation of TGF-β1 seen in mouse mesangial cells and renal glomeruli of mouse models of DN [117, 118]. Mice with deletion of miR192 in vivo were protected from DN [119]. miR192 promoter has also been seen to be regulated by Smads via histone acetylation via TGF-β in renal cells [120, 121]. The downregulation of miR29 correlated with an increase in collagens I, III and IV in ApoE-/- diabetic mouse kidneys in proximal tubuloepithelial cells, mesangial cells, podocytes and TGF-β. miR192 promotes fibrosis with miR29 being anti-fibrotic [120, 122]. A single study, however, showed in patients with diabetes a decrease in miR192 was associated with increased severity of fibrosis and DN; however, this study did not determine normal levels of miR192 in healthy fibrosis [123].

The miRs levels vary from cell type to cell type, and thus, the overall levels in a condition need to be determined before this can be used as a tool to predict progression of DN. Urinary miR levels have been measured together with circulating levels in patients with CKD and may prove to be useful biomarkers of disease [124]. Potential anti-miR target drugs with conventional therapy may be a new approach in the management of DN.

Recently, Pena et al. [103] published an observational study looking at the value of a panel of 28 serum biomarkers on predicting a decline in GFR in 82 patients with a 4-year follow-up. The two statistical models determined a change of <3 ml/min/1.73 m^2^/year with the chosen biomarkers reflecting different pathways previously reported to be involved in DN. Other studies have used urinary biomarkers [125] or a combination of plasma and urinary biomarkers [126]; however, these panels have not been validated or used in a mixed population of T1/T2DM with varying degrees of albuminuria.

Another approach to the selection of biomarkers has recently been reported by Lambers Heerspink et al. [127]. The SysKid consortium performed a comparison of systematic analysis of molecular pathways for DN previously described in the literature with a systematic analysis of the molecular pathways and mode of action of a drug of interest, such as ACEi. The overlapping molecular pathways were used to identify biomarkers similar to both and provide a basis to study subgroups and identify these as phenotype profiling. This novel approach may identify within-groups genetic polymorphisms that determine whether a specific group within the DN spectrum will have a more effective response to the therapy and thereby enhance personalised therapies to precision medicine. This data analysis combines genomic, proteomic, metabolomic and transcriptomic molecular pathways and closely centres around the effects of new therapies to provide biomarkers and maybe useful when monitoring the treatment effect of certain therapies in the future once validated.

The pathogenesis of DN is increasingly accepted as involving multiple mechanisms [24], and in response, there are now a host of novel approaches to DN (Table 5). New therapeutic strategies are emerging with some currently being conducted in small trials with some promising initial results [128–131].

**Table 5:** New therapies for DN

Agent	Target	Studies	Effect
Nrf-2 activator (triterpenoid RTA dh404)	Nrf-2	Animal	Restores Nrf-2 activity decreases oxidative stress
Pyridoxamine dihydrochloride (vitamin B6)	Advanced glycation end product (AGE) inhibitor	Human	Decreases AGE levels, ACR and improves Creatinine
Endothelin 1A antagonist (atrasentan, avosentan)	Endothelin 1A receptor	Animal and human	Reduction in ACR in DN and non-diabetic CKD
Daglutril	Endothelin-converting enzyme and neutral endopeptidase inhibitor	Animal Human—RCT	Anti-fibrotic in animals Reduction in BP irrespective of A2RB in humans.
Pentoxifylline	TNF-α blockade	PREDIAN—human phase 3 trial	Reduction of proteinuria in addition to ACEi/ARB
Doxycycline	Metalloproteinase inhibitor, tetracycline	Human—small RCT	Reduction in ACR while on treatment
Allopurinol	Xanthine oxidase inhibitor	Human—RCT	Reduction in ACR and serum Cr, improves GFR
Silymarin (milk thistle)	antioxidant, TGF-β	Human—small RCT	Reduction in ACR, urinary TNFα and malondialdehyde
Pirfenidone	TGF-β	Small RCT Animal studies	Improved GFR at 1 year—gastrointestinal side effects
Anti-CTGF monoclonal antibody (FG-3019)	CTGF	Animal Human—phase 1	Reduction in ACR in microalbuminurics
Paricalcitol (vitamin D)	Vitamin D	Small RCT—VITAL study	Reduction in ACR in DN. No effect on overall mortality
RS102895	Chemokine receptor CCR2 antagonist	Animal Human—phase 2 (CCX140-B)	Animal—reduction in ACR, improved histological features, decrease oxidative stress with improved glucose tolerance
VEGF antibody antagonist	Vascular endothelial growth factor (VEGF)	Animal	Decrease glomerular hypertrophy, hyperfiltration and albuminuria
Octreotide	Somatostatin agonist	Animal Human	Improved GFR, reduction in ACR, normal renal volume
ACTH gel	Adrenocorticotropic hormone	Human	Reduction in ACR. No effect on renal function

## Novel interventions to tackle the problem

### Antioxidants and anti-inflammatories

Oxidative stress and inflammation have been increasingly described to be active in the progression of CKD, in particular in DN [128, 132, 133]. Nuclear-1-factor (erythroid-derived 2)-related factor 2 (Nrf2) is a transcription factor driving the antioxidant response, impaired in experimental animal models of CKD [134]. Bardoxolone methyl is a synthetic triterpenoid that activates the Nrf2 pathway, restoring antioxidant response. Early human studies with T2DM and CKD stage 3b–4 reported reductions in serum creatinine concentration with bardoxolone methyl for up to 52 weeks [135, 136]. Subsequently, the BEACON trial was designed to determine whether bardoxolone methyl reduced the risk of ESKD or cardiovascular mortality in T2DM with CKD stage 4 [137]. A significant increase in GFR, BP, body weight and urinary ACR was seen in the bardoxolone methyl compared with the placebo group. However, the BEACON trial was terminated following a median follow-up of 9 months after 43 patients receiving bardoxolone methyl developed ESKD and increased cardiovascular morbidity and mortality (from any cause) were observed in the intervention arm when compared to placebo, with “heart failure” being a prominent signal. It is postulated that the side effects arose due to a longer exposure period to the drug in individuals with a more severe form of CKD; however, further detailed analysis is pending. There were suggestions that bardoxolone was toxic in mouse models of DN that were not translated to the initial human study although the improvement in GFR in the initial study remains unexplained [138]. Novel Nrf2 activators are in the pipeline, and future studies will determine their potential benefit in the clinical setting [139–141].

Smaller studies have looked at other targets to determine whether there is any benefit to combined treatment with RAAS blockade (Table 5). Pyridoxamine (vitamin B6) has been shown in animal studies to be a natural inhibitor of AGE with additional reductions in albuminuria and preservation of renal function [142, 143]. Lewis et al. [144] conducted a randomised double-blind placebo-controlled trial in T2DM treated with pyridoxamine for a year, but the results were negative.

Pentoxifylline is a methylxanthine derivative with inhibitory actions against TNF-α. The PREDIAN trial shows significant reductions in proteinuria (*p* < 0.01) and preservation of renal function with pentoxifylline in combination with ACEi/ARB therapy compared to ACEi/ARB therapy alone (*p* < 0.001) in CKD stages 3/4 and T2DM [145].

Silymarin is a herbal drug with antioxidant, anti-inflammatory and anti-fibrotic properties. It is the active constituent of the seed Silybum marianum, otherwise known as milk thistle. In a small study, silymarin reduced albuminuria, urinary TNFα and malondialdehyde—a marker of oxidative stress in patients with DN [146].

Macrophage recruitment into adipose tissue is thought to contribute to insulin resistance. The development of a CCR2/MCP-1 antagonist in animal studies was accompanied by better glycaemic control while decreasing glomerulosclerosis and albuminuria in diabetic mice [147]. More recently, the results of a randomised placebo-controlled study in 89 T2DM normoalbuminuric patients were given the CCR2 antagonist has shown improved glycaemic control while being generally well tolerated [148].

### Anti-fibrotics

Endothelin-1 has established pro-fibrotic effects within the kidney [149] following activation of endothelin-1A receptors leading to renal cell injury, inflammation and fibrosis. Endothelin is raised in CKD with endothelin receptor antagonists reported to ameliorate these effects in experimental models of CKD [150]. Endothelin-1A antagonist (atrasentan) with ACEi/ARB significantly reduced albuminuria in a recent randomised controlled trial in T2DM; however, heart failure and fluid overload were the main side effects limiting this treatment [151]. A novel endothelin antagonist (avosentant) is currently been tested for renal and cardiovascular endpoint in patients with T2DM and proteinuria as add-on to RAAS inhibition.

Daglutril is a combination between endothelin-converting enzyme and neutral endopeptidase inhibitor drugs with potent anti-hypertensive actions that are synergistic with RAAS blockade [152]. Minimal facial and peripheral oedema was thought to arise from the inhibitions of the neutral endopeptidase that increase natriuretic peptides, bradykinin and substance P. This 8-week study showed effects on BP alone. Longer studies will determine any potential renoprotective effect of this drug in diabetes.

The anti-fibrotic pirfenidone inhibits TGF-β in animal models [131]. A small trial showed an improvement in GFR at 1 year, but gastrointestinal symptoms and fatigue were common in this small study [153]. Interestingly, the benefit in GFR was seen using a lower rather than a higher dose of pirfenidone and further studies are required. CTGF has been shown to have an important effect on the development of renal fibrosis in DN [154]. The anti-CTGF monoclonal antibody has been given to 24 microalbuminuric T1DM or T2DM with the effect of reducing albuminuria up to a year later; however, a larger trial is pending [130]. Recently, anti-TGF-β1 therapy failed to slow disease progression in patients with advanced DN in a randomised, double-masked, phase 2 dose-ranging study [155].

Vitamin D deficiency has been shown in animal studies to contribute to the severity of albuminuria, podocyte effacement and increased glomerular basement membrane [156]. Replacement with an active analogue reduces proteinuria, interstitial fibrosis while improving renal function [157, 158]. A meta-analysis of vitamin D analogues in humans has shown a reduction of proteinuria in combination with RAAS blockade using an ACEi/ARB [159].

An increase in the expression of VEGF has been described in renal biopsies with DN and is thought to contribute to albuminuria and glomerulosclerosis [4, 34]. Antibodies against VEGF have shown a reduction in the histological features of DN in diabetic rats with a decrease in albuminuria [160], and may be a potential future treatment.

### Anti-proteinurics

Octreotide is a somatostatin analogue that decreases mesangial expansion and albuminuria in DM animal models [161]. A small study showed an improvement in GFR, ACR and no change in renal biopsy volume in T1DM given octreotide [162], with a further study showing a reduction of albuminuria following 6 months of treatment [163].

Recently, subcutaneous adrenocorticotrophic hormone gel was given to patients with DN with nephrotic range proteinuria; this treatment achieved a significant reduction in proteinuria after 6–12 months which continued for a year following cessation of treatment. Renal function remained stable in these studies [164].

Doxycycline is a tetracycline antibiotic that inhibits matrix metalloproteinases (MMPs). A decrease in proteinuria was seen at 6 months in combination with RAAS blockade and anti-hyperglycaemic agents in a small cohort [165]. The cessation of doxycycline resulted in the return of previous levels of albuminuria. Allopurinol used in asymptomatic hyper-uricaemic patients with DN was seen to reduce albuminuria in 4 months of treatment in this small RCT of 40 patients and could be used as an adjunct with RAAS blockade [166]. More recently, the PERL is underway recruiting patients with T1DM and CKD stages 1–3 to be given allopurinol for a 3-year period, and the results are awaited [167]. However, allopurinol has recently been reported in T2DM to significantly decrease serum uric acid, ACR, serum creatinine and improve GFR compared to the conventional treated arm [168].

## Current limitations and challenges of regulatory requirements for study of late-stage DN

The old adage ignorance is bliss may be applied with the current belief that there is adequate understanding in the field of the pathogenesis of this disease that has been described since Egyptian times [169] and continues to afflict millions. The major limitation with studies in this field fundamentally lies around the lack of a good biomarker to determine disease stage and without establishing the pathophysiology of this disease to a higher-degree primary prevention, and early intervention trials are made incredibly difficult. Establishing whether glomerular processes versus tubular damage versus ROS/other pathway activation occur first will more effectively allow new emerging therapies to be personalised to the individual with monitoring, using a panel of more sensitive markers reflecting underlying damage or activation of established pathways. Further validation of biomarkers arising from the comparison of molecular pathways of certain DN phenotypes whose genetic polymorphisms may make them more susceptible to certain drug therapies may provide a platform for precision medicine in the future.

The emergence of new biomarkers should be considered to run in parallel with albuminuria in trials allowing for increased sensitivity and comparison to the classical ‘normo-, micro- and macroalbuminuria’ while we increase our understanding. Subsequently, biomarkers that have been discovered depicting early or later stages should be employed to allow treatment effects to be determined. The biomarkers should be used to assess the different CKD stages occurring within those with DN and arguably the change of GFR may be a better comparator for new biomarkers. The treatments should then be used to target the area the biomarker suggests is the most likely active cause of damage. Mann et al. [170] recently refer to the benefit of using a roadmap for studies involving a number of different stakeholders such as clinicians, statisticians, patient groups, health insurance and pharmaceutical companies, biobanks and regulatory agencies, to help avoid delays in the discovery and introduction of therapies and biomarkers to the clinic.

Without the roadmap approach, funding inevitably limits long-term studies and emphasises the importance of multicentre trials whereby large groups of people with the same CKD stage can be compared. The additional challenge is that of the different rates of progression affecting those with DN that may be in part influenced by whether patients are T1DM or T2DM. The final common pathway of fibrosis affects both; however, it is well established that the autoimmune T1DM differs from the metabolic phenotype of those with T2DM and insulin resistance rather than autoimmune destruction of the pancreatic β-islet cells. It would seem more prudent to continue to separate trials for type of diabetes or to conduct trials with a subgroup analysis of T1DM and T2DM within the CKD stages to ascertain whether the results and biomarkers behave in the same manner or differ, thereby making markers more robust while more importantly allowing clarity as to what population benefits from a particular new therapy.

*Where does this leave the researcher?*—to investigate the pathophysiology and markers of disease to determine new therapeutic targets with this understanding. Great attention to differences seen in translational treatments is important in view of the recent experience with bardoxolone.

*Where does this leave the clinician?*—to be aware of changes occurring in the field and to actively enrol appropriate patients into trials where new therapies are being monitored with more sensitive markers. Both researcher and clinician need to urgently review the current primary endpoints of: doubling of serum creatinine, ESKD or death used in this chronic disease especially with early intervention trials. For trials conducted in late-stage DN, all patients should be monitored in specialised DN clinics. In addition, clinicians need to establish within their countries health service infrastructure how patients may be followed up with either dedicated DN clinics or primary care facilities to record how effective therapies are following the completion of initial funding for the trial period. We would challenge the cessation of follow-up for these patients until we are able to determine outcome and would advocate a national database for DN. Larger multicentre trials will naturally enhance our understanding in this field and should be increasingly considered in view of the worldwide increasing incidence of this devastating disease.

*More importantly*, *where does this leave the patients?*—in a new era, an awakening leading to personalised treatment goals according to the progression or stability of their disease with the hope of preventing progression to ESKD.

## Final conclusions and future strategies for clinical research

RAAS blockade alone is insufficient in preventing the progression of diabetic kidney disease in many patients. Good glycaemic control is important in reducing mortality in this cohort of patients; however, very tight glycaemic control may be deleterious [171]. No benefits have been reported for aspirin in the primary prevention of cardiovascular mortality in patients with diabetes; however, it is recommended in those who have had a cardiovascular event. Statins may reduce major cardiovascular events without affecting the progression of renal disease.

There are clear and urgent needs to improve our understanding of the pathogenesis of DN, identify new interventional therapeutic targets and develop new markers of disease progression. The new targets for intervention need to be tested using a melange of old and new markers of disease progression to maximise the chance of detecting useful biological signals. New therapeutics will become available presenting the challenge of when to begin these treatments and how to tailor treatments to the individual. Some of these agents such as vitamin B6, pentoxifylline and vitamin D are readily available and may be introduced to current therapy strategies. Whether any of these novel approaches can usefully reduce mortality remains to be determined.

This review emphasises the need for physicians to look anew at treatment strategies for this high-risk group and perhaps employ the use of multiple treatment strategies beyond that of RAAS blockade. We challenge the current renal endpoints used and how we continue to conduct trials. The need for new therapeutic agents, coupled with refined and more sophisticated trial renal endpoints using new biomarkers, is both obvious and urgent requiring our community to reunite in order to advance treatment strategies in this field.
